# Ovarian torsion in a 2-year-old girl: A case report

**DOI:** 10.18502/ijrm.v21i4.13274

**Published:** 2023-05-08

**Authors:** Anahita Haghjoo, Rahil Haghjoo, Marzieh Rahimipour

**Affiliations:** ^1^Department of Obstetrics and Gynecology, Jahrom University of Medical Sciences, Jahrom, Iran.; ^2^Department of Anatomical Sciences, Jahrom University of Medical Sciences, Jahrom, Iran.

**Keywords:** Ovarian torsion, Child, Laparotomy.

## Abstract

**Background:**

Ovarian torsion (adnexal torsion) is a rare event in pediatric patients which is primarily managed by pediatric general surgeons.

**Case presentation:**

This study presents a case of ovarian torsion in a 2-yr-old girl with a history of episodic lower abdominal pain, nausea, and vomiting for 2 days. Her physical examination was normal except for mild tenderness in the lower abdomen with no palpable mass. A color Doppler ultrasound was performed for further investigation, and an ovarian torsion was reported without sonographic signs of intussusception and acute appendicitis, so she underwent laparotomy. A relatively complete torsion was observed in the left ovarian pedicle. Initially, the left ovary and fallopian tube had a dark appearance, and 10-15% of the ovarian tissue was still normal. Detorsion of ovary was done and it was decided to preserve the ovary. After about 20 min, the color of ovary and fallopian tube returned to relatively normal, indicating normal blood flow. The patient was discharged 2 days later because a follow-up color Doppler ultrasound showed normal ovarian blood flow.

**Conclusion:**

The possibility of ovarian torsion must be considered in all female infants with suspicious abdominal pain.

## 1. Introduction

Abnormal twisting of the fallopian tube and ovary around the ovarian pedicle results in ovarian torsion (1). In most cases, ovarian torsion occurs during the reproductive years, while idiopathic torsion is a rare event within the pediatric age group (2). In children, adnexal torsion can occur at different ages, but > 52% of cases occur between the ages of 9 and 14, with an average of 11 yr. ovarian torsion is also rare in neonates. It occurs in 16% of girls < 1 yr (3, 4).

The signs and symptoms of ovarian torsion, like lower abdominal pain, fever, nausea, and vomiting, are usually nonspecific. Tenderness in the lower abdomen and sometimes a mass may be revealed by clinical examination. Differential diagnosis includes intussusception, infantile colic, appendicitis, acute gastroenteritis, constipation, mesenteric lymphadenitis, ureteric colic, and urinary tract infection (5).

Adnexal torsion is a clinical diagnosis. When the clinical features, patient history, and/or imaging are suspected of adnexal torsion, immediate exploratory surgery makes the final diagnosis. In the pediatric population, the best diagnostic and therapeutic approach is laparoscopic surgery.

The persistence of pain for more than 10 hr is linked to an increased rate of tissue necrosis. So, fast diagnosis and operation can prevent irreversible adnexal damage, salvaging the adnexal torsion, and increasing the success of ovarian preservation (6-9). This clinical case report presented a 2-yr-old girl with left ovarian torsion as a rare case.

## 2. Case Presentation

The case is a 2-yr-old girl complaining of episodic lower abdominal pain, nausea, and vomiting for 2 days. Her parents visited the emergency room in Jahrom Motahari hospital, Jahrom, Iran and they were advised for further observation at home due to absence of any physical exam findings. However, since abdominal pain, nausea, and vomiting (4 times) continued until the night, they visited the hospital again. At admission, she looked pale and lethargic with vital signs as follows: BP: 90/60, PR: 100, T: 36/5, RR: 24, O2 sat: 96%.

**Figure 1:**
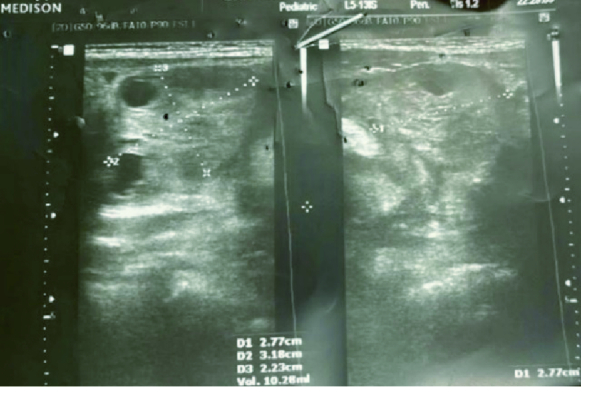
The left ovary is prominent in size with no definite flow.

**Figure 2:**
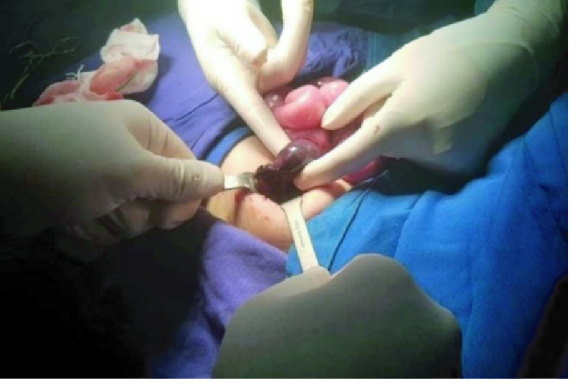
The large adnexa, which is dark due to torsion of the pedicle.

**Figure 3:**
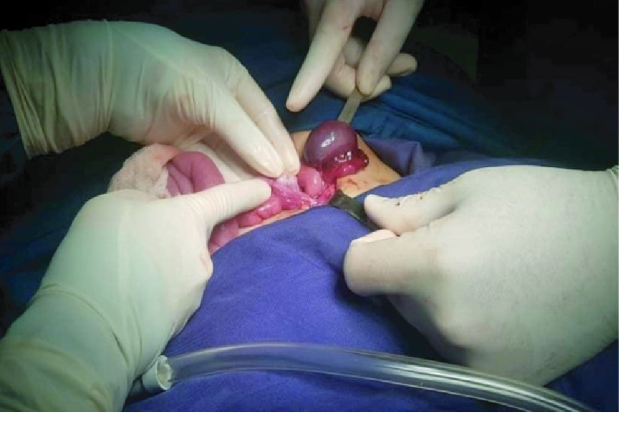
The ovary and fallopian tube after detorsion: The color of the ovary and fallopian tube returned to normal, indicating normal blood flow.

Her physical examination was normal except for mild tenderness in the lower abdomen with no palpable mass. Moreover, leukocytosis was noted in complete blood count. So, an urgent abdominopelvic ultrasound was performed, demonstrating a prominent left ovary size of 33 × 42 mm with no definite vascularity that was highly suspicious for ovarian torsion (Figure 1). No sonographic sign of intussusception and acute appendicitis were observed. Eventually, she underwent laparotomy due to ovarian torsion. A relatively complete torsion was observed in the left ovarian pedicle. Ovarian detorsion was done and decided to preserve the ovary even though the left ovary and fallopian tube had a dark appearance and 10-15% of the ovarian tissue was normal (Figure 2). After about 20 min, color of the ovary and fallopian tube returned to normal, which indicated normal blood flow (Figure 3). The patient was discharged in stable condition 2 days after the surgery because the color Doppler ultrasound showed normal ovarian blood flow.

### Ethical considerations

Ethical considerations were maintained during this study, and written consent was taken from the patient's parents for this presentation.

## 3. Discussion

Ovarian torsion commonly occurs during childbearing age and postmenopausal age (5). It is estimated to be about 5 per 100,000 in females between 1 and 20 yr old (1). There are several risk factors associated with ovarian torsion, such as ovarian mass (especially dermoid) or cyst, previous torsion or surgery, polycystic ovary, tubal sterilization, and pregnancy (10, 11). In the pediatric patient, about 51-84% of adnexal torsions occur due to adnexal pathology, which includes follicular or hemorrhagic ovarian cysts, cystic teratomas or dermoid, and less frequently cystadenomas, para ovarian cysts, and hydrosalpinx (8, 12).

Physical examination findings, such as normal temperature, low-grade fever, and/or mild tachycardia, are typically nonspecific to adnexal torsion. Acute pelvic or abdominal pain with variable duration (days to months) is the most common symptom, and it is often isolated to one side. Also, it can be mild or intense, non-radiating, constant, or intermittent (depending on whether the torsion is partial or complete). In addition, symptoms such as flank pain, nausea and vomiting due to peritoneal reflexes, and anorexia are usually associated with adnexal torsion. In patients with adnexal torsion, most laboratory findings are normal. However, a slight leukocytosis can sometimes occur (13). Lower abdominal pain, nausea, vomiting, mild tenderness in the lower abdomen with no palpable mass, and leukocytosis were diagnosed in this patient.

Clinical diagnosis of ovarian torsion must be differentiated from hemorrhagic ovarian cyst rupture, appendicitis, gastroenteritis, and kidney stone (8, 13, 14). Moreover, adnexal torsion is often difficult to diagnose because of the vague and variable clinical signs and nonspecific imaging findings. To evaluate blood flow to the ovaries in pediatric and adolescent patients, pelvic ultrasonography with color Doppler is the common and accurate diagnostic imaging of adnexal torsion (14, 15). Therefore, ultrasonography is usually enough for diagnosis (5).

Computed tomography and magnetic resonance imaging are not the first-line imaging modalities in adnexal torsion, but they can be used to assess the structure if ultrasound is not available or if the differential diagnosis remains with other cases such as appendicitis (14). Delay in diagnosis of ovarian torsion commonly leads to loss of the involved ovary and other complications (5).

Ovarian torsion can be treated with detorsion or oophorectomy. Detorsion is a safe and effective treatment for ovarian torsion, and it may be more optimal for all premenopausal women because of the high rates of ovarian preservation and low risk of complications (16). Recent evaluation of pediatric patients in the national inpatient sample showed that 15%, 6%, and 78% of cases underwent detorsion, detorsion with oophoropexy, and oophorectomy alone, respectively (17). Another case report presented ovarian torsion and histopathological disorders, including cystic lesions with extensive calcification in a 4-month-old baby undergoing oophorectomy (5).

Although ovarian torsion is rare in children, early diagnosis is critical because it can save the ovary and prevent psychological trauma to the parents and child. Therefore, the possibility of ovarian torsion must be considered in all female infants with suspicious abdominal pain.

##  Conflict of Interest

The authors have no conflict of interest relevant to this article.
